# Oxygen in the tumor microenvironment: effects on dendritic cell function

**DOI:** 10.18632/oncotarget.26608

**Published:** 2019-01-25

**Authors:** Laurent M. Paardekooper, Willemijn Vos, Geert van den Bogaart

**Affiliations:** ^1^ Department of Tumor Immunology, Radboud Institute for Molecular Life Sciences, Radboud University Medical Center, Nijmegen, The Netherlands; ^2^ Department of Molecular Immunology, Groningen Biomolecular Sciences and Biotechnology Institute, University of Groningen, Groningen, The Netherlands

**Keywords:** tumor microenvironment, dendritic cells, hypoxia, reactive oxygen species, extracellular vesicles

## Abstract

Solid tumors grow at a high speed leading to insufficient blood supply to tumor cells. This makes the tumor hypoxic, resulting in the Warburg effect and an increased generation of reactive oxygen species (ROS). Hypoxia and ROS affect immune cells in the tumor micro-environment, thereby affecting their immune function. Here, we review the known effects of hypoxia and ROS on the function and physiology of dendritic cells (DCs). DCs can (cross-)present tumor antigen to activate naive T cells, which play a pivotal role in anti-tumor immunity. ROS might enter DCs via aquaporins in the plasma membrane, diffusion across the plasma membrane or via extracellular vesicles (EVs) released by tumor cells. Hypoxia and ROS exert complex effects on DCs, and can both inhibit and activate maturation of immature DCs. Furthermore, ROS transferred by EVs and/or produced by the DC can both promote antigen (cross-)presentation through phagosomal alkalinization, which preserves antigens by inhibiting proteases, and by direct oxidative modification of proteases. Hypoxia leads to a more migratory and inflammatory DC phenotype. Lastly, hypoxia alters DCs to shift the T- cell response towards a tumor suppressive T_h_17 phenotype. From numerous studies, the concept is emerging that hypoxia and ROS are mutually dependent effectors on DC function in the tumor micro-environment. Understanding their precise roles and interplay is important given that an adaptive immune response is required to clear tumor cells.

## INTRODUCTION

When solid tumors grow, the oxygen demand increases rapidly while there is insufficient vascularization. This causes the tumor to become hypoxic at the tumor core and the edges of the invasive front [1]. Due to the lack of oxygen, ATP is mostly produced via a high rate of anaerobic glycolysis; this is called the Warburg effect. The Warburg effect leads to lactic acid fermentation in the cytosol and increased oxidative stress in the form of H_2_O_2_ and other radicals. H_2_O_2_ activates the transcription factor Nrf2 which further upregulates glycolysis-related genes and further contributes to the Warburg effect [2, 3]. ROS is important for tumor growth via the kinase AMPK. AMPK can suppress cell proliferation via cell cycle arrest [4] and its activation depends on reduction of cysteine residues by thioredoxin (Trx) at the catalytic subunit alpha [5, 6]. However, these sites can be oxidized by ROS to inactivate AMPK, promoting tumor cell proliferation. The increase in oxidative stress also translates to the tumor microenvironment (TME). The TME comprises the tumor cells itself and the stromal cell compartment directly surrounding it, containing blood vessels, cells from the immune system, fibroblasts and extracellular matrix [7, 8].

**Figure 1:**
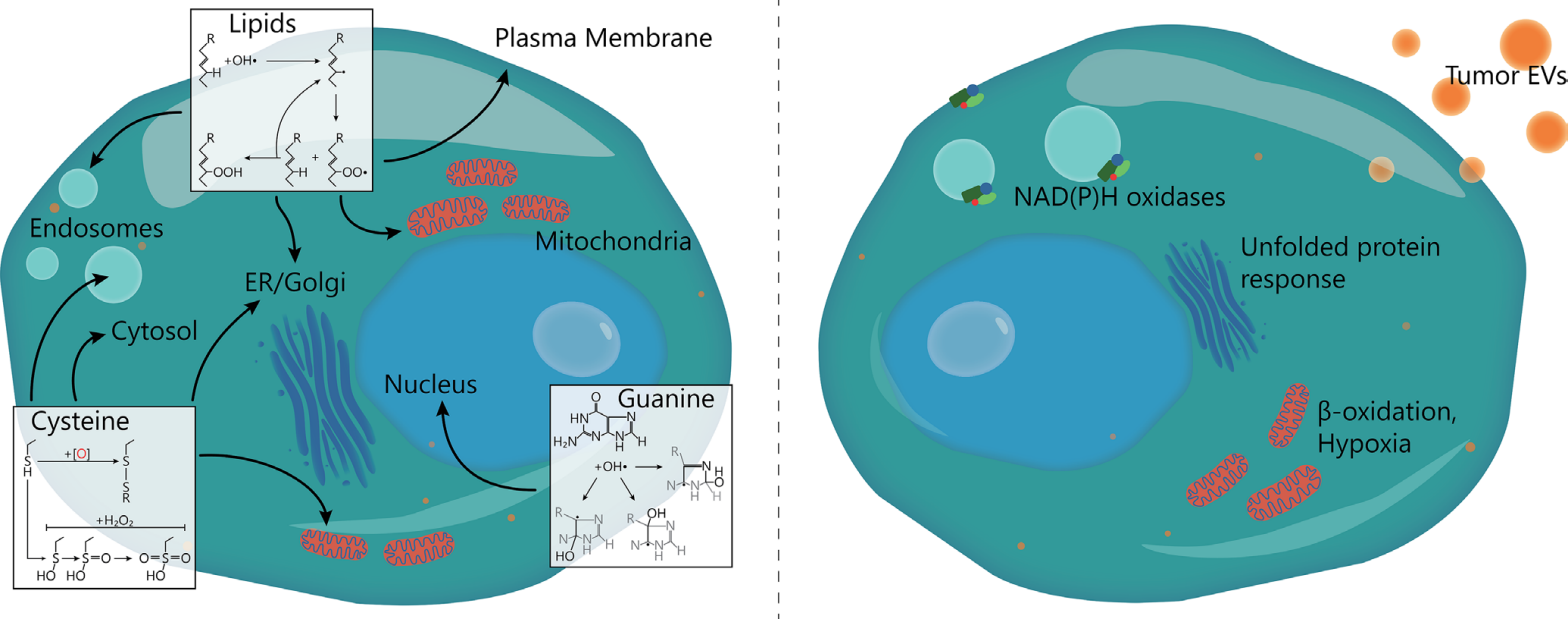
Targets and sources of ROS in DCs (Left) ROS can attack both mono- and poly-unsaturated lipids in membranes, causing endosomal leakage and loss of pH and electron gradients. Cysteine residues on proteins can oxidize, resulting in the formation of disulfide bridges or a stepwise oxidation to sulfonic acid. This can activate redox-sensitive signaling factors, but also block enzymatic activity or cause protein misfolding. Finally, both free and DNA-helix incorporated guanine nucleotides can oxidize, leading to GC-TA or GC-CG transversion mutations. (Right) Sources of ROS for DCs in the TME: increased NOX (NAD(P)H oxidase) activity, ER stress due to the unfolded protein response, β-oxidation of fatty acids, abrogated electron transfer in mitochondria and uptake of ROS-containing tumor-derived EVs.

In cancer cells, there are multiple sources of ROS (Figure 1). Oncogenic mutations (e.g., in Ras, Myc and p53) can cause mitochondrial dysfunction and increased leakage of ROS. This leakage occurs mostly at complex 1 or complex 3 of the respiratory chain, where electrons from NAD(P)H or FADH_2_ react with oxygen to form superoxide anion. Second, ROS is formed enzymatically by NAD(P)H oxidases (NOX), which can be activated by various growth factors, such as VEGF and angiopoietin which are often upregulated in cancer [9, 10]. Third, exogenous radiation (e.g., UV light, radiotherapy) can cause the production of ROS [11]. Fourth, ROS are by-products of cellular metabolism such as from protein folding and beta-oxidation of fatty acids in the mitochondria and peroxisomes [2, 12]. Last, intracellular enzymes, such as xanthine oxidase, cyclooxygenases, cytochrome p450 enzymes, lipoxygenases and nitric oxide synthetase, generate ROS as a metabolic byproduct [11, 13]. Tumor cells thus have to cope with comparatively high intracellular levels of ROS and they do this by upregulating antioxidative systems such as Trx, peroxiredoxin (Prx), catalase, superoxide dismutases (SOD) and generation of NAD(P)H [13]. Against superoxide anion specifically, dismutation to H_2_O_2_ is the primary protective mechanism and this can occur enzymatically by SOD or spontaneously in acidic environments. Subsequently, H_2_O_2_ can then be degraded into water and oxygen by catalase [14].

In addition to cancer cells, ROS-producing immune cells of myeloid origin are present in the TME [15]. These include DCs (see below), but are mainly macrophages that have differentiated from circulating monocytes [16] and, depending on the stimulus, can develop towards more cytotoxic or immunosuppressive phenotypes [17]. However, tumor-associated macrophages in the TME are usually immunosuppressive, as they produce IL-10 and transforming growth factor β [18] and recruit regulatory T cells via CCL22 [19]. ROS produced by these cells were found to be detrimental for cancer progression, as increased H_2_O_2_ secretion by macrophages was shown to be a sufficient trigger for both tumor initiation and development of epithelial cancer [20]. This was further exacerbated by H_2_O_2_-mediated induction of TNF-α and TNF receptor 1 transcription, which lead to recruitment of more H_2_O_2_-secreting macrophages. Additionally, ROS production by the monocyte precursors of these macrophages was shown to be a strong determinant for development towards an immunosuppressive phenotype [21].

Hypoxia and oxidative stress influence multiple functions of the cancer cells, such as angiogenesis, cell proliferation and apoptosis, and thereby can promote tumorigenesis. H_2_O_2_ promotes angiogenesis by activating the transcription factors NF-κB and AP-1, leading to activation of VEGF transcription factors NF-κB and AP1 [22], VEGF secretion [23] as well as VEGF receptor 2 transcription [24]. In addition, H_2_O_2_ can also activate VEGF receptor 2 in a ligand-independent manner via Src kinases [25]. VEGF activates NOX and thereby leads to the generation of more ROS, forming a positive feedback loop, and making NOX a potential therapeutic target for inhibition of angiogenesis [2, 9]. A major mechanism by which ROS affects physiological processes is by the formation of disulfide bonds. For example, H_2_O_2_ modifies the thiolates of cysteine residues in redox sensitive proteins [13] and particularly zinc-bound cysteines perform oxidative stress sensing [26]. Zinc-bound cysteines are present in zinc finger transcription factors, for example the GATA family of transcription factors and Krüppel-like Factor 2, both of which are involved in ROS-mediated signaling pathways [27–30]. Lastly, ROS influence apoptosis induced by the dimerization of the kinase ASK1 with TRAF2, which causes apoptosis via activation of the kinases JNK and p38 MAPK. This dimerization is induced by H_2_O_2_, but blocked by thioredoxin (Trx) [31, 32]. Trx is bound to ASK1 by a disulfide bond at the N-terminal domain of ASK1, leading to ubiquitination and proteosomal degradation of ASK1. High levels of H_2_O_2_ counteract the functioning of Trx and cause the release of Trx from ASK1, because of the formation of an intramolecular disulfide bond [33].

It is not completely understood how hypoxia and ROS in the TME influence infiltrating immune cells, which is the focus of this review. Especially DCs play a major role in generating anti-tumor immunity, due to their ability to activate naive T cells. After encountering an antigen, DCs can maturate and migrate to the lymph nodes to present processed antigens to T cells. The ability of DCs to (cross-)present antigens and activate T cells is influenced by the inflammatory environments that the cells encounter [34]. Since DCs encounter a variety of environments that differ in oxygen tension and ROS levels during antigen uptake and migration to the lymph nodes, it is likely that these environments affect the final immune response. The aim of this review is to provide a comprehensive overview of the effects of TME-associated oxidative stress on DCs.

### ROS entry in DCs

Solid tumors are frequently invaded by DCs and other immune cells, which hence are exposed to the hypoxia and radicals in the TME. ROS may affect the plasma membrane composition of invading immune cells through oxidation of both the lipid bilayer and of membrane proteins [35]. However, to affect intracellular processes, ROS have to traverse the plasma membrane. Many species of ROS, such as superoxide anion, carry a free electron and cannot efficiently traverse the apolar lipid bilayers. However, either spontaneously or catalyzed by the abundant SODs, superoxide anion can dismutate to H_2_O_2_ which is uncharged and does not carry a free electron. H_2_O_2_ has a lower reactivity compared to ROS species such as superoxide anion and hydroxyl radicals and this makes H_2_O_2_ relatively stable and also allows it to diffuse through membranes and to enter the nucleus to cause DNA damage [11]. Its relatively high stability even allows H_2_O_2_ to signal between different cells [13]. These properties allow H_2_O_2_ to increase the redox potential of the TME. H_2_O_2_ cannot only passively diffuse through lipid membranes, but also enter cells through the aquaporin channel AQP8, as shown by heterologous expression in yeast [36], and through AQP1, 3 and 8 in a human leukemia cell line [37]. Both immature and monocyte-derived dendritic cells express AQP3, 7 and 9 and mature DCs express AQP7 and 9 [38, 39], suggesting that H_2_O_2_ can enter DCs via these channels. Other aquaporins might be involved as well, as the homologs AQP7, AQP9, AQP10, AQP12A and AQP12B are all expressed by human immune cells [40]. Superoxide anion can enter endothelial cells by diffusion through the Cl^−^ channel-3 (Clc3) [41] and might also enter immune cells via this channel, as it is expressed in human macrophages and peripheral blood mononuclear cells [42, 43]. However, there is no experimental evidence yet that these channels mediate entry of H_2_O_2_ and superoxide anion in DCs.

### ROS affect DC maturation

ROS are directly implicated in DC maturation by the activation of p38-MAPK and ERK1/2. DCs treated with 1-fluoro-2,4-dinitrobenzene, a skin sensitizer which is perceived as a danger signal by DCs, showed increased ROS production and activation of p38-MAPK via an unknown mechanism [44]. In line with ROS promoting DC activation, H_2_O_2_-treated human peripheral blood DCs were more efficient in promoting T cell proliferation and showed an upregulation of cell surface molecules MHC-II, CD40 and CD86, all important components of T cell activation [45]. The mechanism by which ROS promote DC maturation is not known.

ROS can also reduce DC maturation via ER stress. Danger signals such as 1-fluoro-2,4-dinitrobenzene cause accumulation of misfolded proteins in the cell, leading to ER stress and an increase in mitochondrial ROS production [44]. Oxidative stress can also affect ER function by disturbing Ca^2+^ homeostasis [44]. The accumulation of misfolded proteins activates the unfolded protein response, aimed at restoring normal cell function by halting translation, degradation of misfolded proteins, and increasing expression of chaperone proteins. This study observed that ROS affected the PERK-eIF2α-ATF4 axis of the unfolded protein response, which led to a short-term block of CD86, IL-1β and IL-12B expression in THP-1 monocytes. However, these genes were upregulated at later time points, indicating a pro-inflammatory response. ER stress is also caused by lipid peroxidation products that follow increased ROS production, such as 4-hydroxynonenal (4-HNE). This aldehyde readily forms protein adducts due to its reactivity with thiols and amine groups [46], which can trigger the unfolded protein response. Additionally, 4-HNE was shown to form adducts with ER-resident chaperone proteins [47], leading to increased activation of ER stress transcription factor XBP1 in ovarian cancer-associated DCs [48]. This in turn inhibited anti-tumor immunity via accumulation of lipid bodies in the DC, which blocks translocation of MHC-I to the cell surface [48, 49]. As 4-HNE is relatively stable and able to diffuse through membranes, DCs may even internalize 4-HNE from the TME [50, 51]. Likewise, malondialdehyde, another common lipid peroxidation product, also forms protein adducts which are shown to be strongly auto-immunogenic, which may hamper specific anti-tumor responses [52–54].

### Effects of ROS on antigen presentation

ROS influence the ability of DCs to cross-present antigens to CD8^+^ cells [55]. Upon activation of Toll-like receptors (TLRs), NOX2 is activated in the DCs and produces large amounts of superoxide anion in endo/phagosomes [56–58]. This increases the endo/phagosomal pH through the consumption of protons by the dismutation of superoxide anion to H_2_O_2_ [55, 56]. The increased pH impacts cross-presentation through antigen conservation, as lysosomal hydrolases with acidic pH optima are less activated [55]. In addition, ROS can affect the activity of the endosomal V-ATPase by formation of a disulphide bond between cysteine residues located within the nucleotide-binding subunits, leading to its inactivation and reduced acidification of the endosomal lumen [56, 59]. Endo/phagosomal proteases like cathepsins are modified in a similar fashion, leading to altered epitopes for both MHC-I and MHC-II [60–62]. ROS can also induce the release of antigen from phagosomes into the cytosol by causing leakage of antigens through lipid peroxidation of the endo/phagosomal membranes, and this can promote antigen cross-presentation [57, 63].

However, the effect on antigen presentation depends on the cellular site of ROS production, as a study in aged mice suggested an inhibitory role for mitochondrial ROS in cross-presentation by bone marrow-derived DCs [64]. DCs from aged mice (16–20 months) show signs of mitochondrial dysfunction, leading to increased ROS production compared to DCs from young mice (2–3 months). Scavenging ROS partially improved the cross-presentation efficiency of the DCs from aged mice [64]. This finding indicates that, although DCs actively use endo/phagosomal ROS to enable cross-presentation, a general increase in the environmental redox potential could also hamper cross-presentation.

### Extracellular vesicle release by tumor cells

A recently identified source of ROS in the TME are EVs released by tumor cells. EVs contain many different compounds, including ROS as shown by flow cytometry with a fluorescent ROS probe [58]. ROS was also found in EVs derived from hypoxic/reoxygenated human umbilical vein endothelial cells [65]. However, the source of EVs in the TME is still controversial, and it is debated whether cancer cells can dictate their content or they are only membrane blebs or necrotic cell bodies [66]. There is some evidence for controlled release of EVs in a process involving the endosomal sorting complex [67]. This complex generates the intraluminal vesicles of multivesicular bodies by bulging the membrane inwards onto the lumen of late endosomes. When multivesicular bodies fuse with the plasma membrane, these intraluminal vesicles are released as EVs [68, 69]. However, other mechanisms of EV formation have also been proposed, such as direct shedding from the plasma membrane [70, 71]. EVs of intracellular origin are often called exosomes, while EVs shed from the plasma membrane are called microvesicles. However, this nomenclature and the methods to discriminate between various sources of EVs are not yet standardized, therefore we will use the general term EVs in this review [66].

Several studies showed that hypoxia increases the release of EVs by various types of tumor cells, thereby suppressing the immune response [72, 73]. In a study on breast cancer cells, it was found that the transcription factor HIF-1α is responsible for this increase in EV production, since the release of EVs was blocked upon silencing of HIF-1α [73]. A second study on breast cancer cells came to a similar conclusion, and found that cells exposed to hypoxia overexpressed the small GTPase RAB22A in a HIF dependent manner, leading to increased formation of EVs in breast cancer [74]. The cargo of EVs is possibly influenced by hypoxia as well, as the level of the micro-RNA miR-210 is elevated in EVs upon hypoxia [73]. Transcription of miR-210 is mediated by HIFs and it has target genes involved in cell survival, angiogenesis and metabolism [75].

### Effects of extracellular vesicles on DCs

EVs influence immune cells and for instance may control macrophage differentiation towards an immunosuppressive phenotype [76], but also might exert effects on DC function. Multiple mechanisms are suggested for the uptake of EVs by recipient cells: fusion, receptor-ligand interactions and endocytosis. The uptake of EVs by DCs is mediated by several factors, including CD11a, intracellular adhesion molecule 1, phosphatidylserine and milk fat globule E8 on DCs, and tetraspanins CD9 and CD81 on EVs [77]. Glycosylation is also involved in targeting EVs, as uptake of EVs derived from Jurkat T cells by myeloid DCs was inhibited by blocking Siglec-1 [78]. Siglec-1 preferentially binds to α2,3-linked sialic acids which decorate proteins on the surface of EVs. Furthermore, EV uptake by DCs involves interaction between LFA-1 and C-type lectin receptors like DEC205 on DCs with CD54 or various glycoproteins on the EVs [79].

After uptake, the ROS present in EVs might affect DC function. EVs derived from ovarian cancer cells promote antigen cross-presentation in DCs via ROS-mediated phagosomal alkalization [58], although in this study the effects of other EV components cannot be completely excluded. Moreover, it is unclear whether EV-derived ROS are directly responsible for ROS accumulation inside the phagosomes, or whether this is the result of ROS producing enzymes carried by the EVs [80] or other ROS-inducing components [81]. Besides changing the phagosomal pH, it is proposed that EVs induce antigen-specific tolerance in DCs. Tumor EVs contain antigens and EVs taken up by immature DCs are shown to inhibit the maturation of DCs [82]. In this study, EVs were internalized by mouse CD11c^+^ cells, which subsequently downregulated expression of the maturation markers MHC-II and CD86, while levels of the anti-inflammatory cytokine transforming growth factor β1 were elevated in these DCs. This steered CD4^+^ T cells towards a regulatory phenotype, capable of suppressing B cell responses. The uptake of EVs by DCs can however also promote CD8^+^ T cell responses, benefitting antitumor immunity, because mature DCs pulsed with EVs expressed MHC-I, MHC-II, CD40, CD54 and CD80 at higher levels than controls, while immature DCs pulsed with EVs did not respond [79]. In fact, mice bearing BL6-10_OVA_ tumor cells were able to clear their tumors following adoptive transfer with mature DCs pulsed with OVA-containing EVs, while DCs pulsed with soluble OVA only reduced tumor growth temporarily [79]. These EVs might potentially contain ROS and tumor material which induces ROS and danger-associated molecular pathogen signaling via pattern recognition receptors. This would explain the more efficient cross-presentation of EV-derived antigens compared to soluble antigens [58], as ROS are a major factor in upregulating cross-presentation [55, 57, 60, 61, 83]. However, the effects of EV-containing ROS on antigen presentation are difficult to discern from the effects of other EV components, such as micro-RNAs.

The molecular mechanisms by which EVs affect DC maturation and the role of EV-encapsulated ROS in this process are still largely unknown. In murine CD11b^+^ myeloid DC precursors, tumor EVs inhibited the differentiation of DCs via enhanced expression of interleukin 6 (IL-6) [84]. After EV uptake, CD11c expression was significantly lowered and IL-6 expression was higher than in the control cultures. Precursor DCs were able to differentiate in the CD11c^+^ phenotype, but these DCs were less able to mature as measured by analysis of the expression of the co-stimulatory molecules CD86 and CD80. The expression of CD86 and CD80 was significantly lower and correlated with the concentration of EVs added to the culture [84]. When DC precursors were isolated from IL-6 knockout mice, DC maturation was not inhibited, suggesting that the inhibition is mediated by IL-6. The authors observed similar effects on differentiation of human CD14^+^ monocytes into monocyte-derived DCs after stimulation with isolated EVs from the MDA-MB-231 breast cancer cell line. In conclusion, several immunologically relevant effects of EVs have been described, many of which are likely to depend at least partly on EVs containing ROS. However, the effects of other compounds present in EVs cannot be excluded and this requires further investigation.

### The TME is hypoxic

As mentioned above, a major cause for the generation of ROS in the TME is a disturbed metabolism of the cancer cells. Since ROS generation consumes molecular oxygen, this increases the hypoxic conditions caused by the poor vascularization frequently associated with tumors [85]. A major signaling component downstream of hypoxia is the HIF family of transcription factors, consisting of HIF-1α, HIF-2α and HIF-1β. At normoxic conditions, the alpha subunit is targeted for degradation via hydroxylation by prolyl hydroxylase domain enzymes and factor inhibiting HIF-1α. Upon hydroxylation, HIF-1α and HIF-2α bind to the von Hippel–Lindau tumor-suppressor protein, allowing ubiquitination and ultimately proteasomal degradation [86–88]. The hydroxylation reaction requires oxygen and therefore cannot efficiently occur under hypoxic conditions, resulting in the accumulation of the HIF-1α and/or HIF-2α subunits, allowing them to dimerize with HIF-1β and translocate to the nucleus. Here, the heterodimer binds to the hypoxia responsive element (HRE) in the promotor region of target genes [89–91]. Many of the HIF target genes are involved in angiogenesis and in erythropoiesis, and HIF also promotes glycolysis by upregulating expression of plasma membrane glucose transporters and inhibiting pyruvate dehydrogenase kinase, which blocks the translocation of pyruvate to the tricarboxylic acid cycle [92, 93].

### Hypoxia alters DC differentiation and maturation

The effects of hypoxia on the differentiation and maturation of DCs are quite well studied, although there is little consensus between studies. For example, expression of MHC-II is mostly reported to decrease in hypoxic environments [94–99], but the opposite [100] or no effect [101, 102] have been reported as well. The same holds for DC maturation markers like CD80, CD83 or CD86, where several studies found no effects [99, 101, 102], but upregulation [100] and downregulation [95–97, 103] were reported as well. Hypoxic alteration of MHC-I expression is less well studied, however HIF-1α activity is implied in upregulating MHC-I expression [104]. These contradicting results likely arise from the complex interplay of ROS and hypoxia signaling with immune cell activation pathways. In particular, hypoxia is capable of altering TLR signaling [105] and subsequently leads to altered cytokine secretion patterns [95, 100]. Expression of TLR4 [106] and its downstream kinase MAP3K8 (also known as Cot or TPL-2) are upregulated by hypoxia [90, 107]. This leads to an hypoxic potentiation of TLR4-mediated secretion of TNF-α in human monocyte-derived DCs [107]. In line with this, hypoxia has also been found to increase secretion of other pro-inflammatory cytokines such as IL-6, IL-8 and IL-1β in primary human macrophages and osteoclasts [95, 108–110]. So, while hypoxia itself does not cause ROS formation, it can trigger inflammation which in turn promotes ROS formation. Since ROS formation consumes oxygen, it causes additional hypoxia, resulting in a feedback loop.

Hypoxia skews immature DCs towards a highly mobile phenotype by upregulating genes involved in cell motility [111]. The chemokine receptors CCR2, CCR3, CCR5, CX3CR1, C5R1 and FPR3 are upregulated upon hypoxia, while expression of chemokines CCL26, CCL24 and CCL14 are inhibited by hypoxia [98, 112]. This suggests that immature DCs differentiated in a hypoxic TME migrate away towards normoxic tissues, potentially suppressing anti-tumor immunity. Moreover, a variety of tumors secrete elevated levels of prostaglandin E2, which is a strong migratory stimulus for immature DCs [112]. Prostaglandin E2 is generated by cyclooxygenases, many of which are HIF target genes [90, 108]. In contrast, mature DCs downregulate expression of CCR7 in hypoxic conditions. CCR7 is the chemokine receptor which signals DCs to migrate to draining lymph nodes in response to lymph node derived chemokines [98]. This observation is in line with the poor chemotactic ability of hypoxic mature DCs in response to the lymph node chemokine MIP-3β. Thus, evidence suggests that the hypoxic TME promotes the expulsion of immature DCs from the tumor, whereas the migration of mature DCs to the lymph nodes is reduced.

The effects of hypoxia on DC maturation might well be transient. Murine DCs cultured under low oxygen tensions expressed lower levels of MHC-II, CD80 and CD86 compared to DCs under normoxic conditions [96]. However, reoxygenation of hypoxia-differentiated DCs restored the levels of these maturation markers, suggesting that once these DCs migrate towards the lymph nodes they can regain full functionality [96]. Finally, hypoxia affects antigen uptake as it decreased the phagocytic capacity of immature DC compared to DCs cultured under normoxic conditions [98]. In conclusion, hypoxia can both increase and decrease DC maturation, likely depending on the presence of ROS and other inflammatory stimuli in the TME. Moreover, hypoxia can promote immune tolerance, as it stimulates migration of immature DCs out of the TME while restricting migration of mature DCs to prevent T cell activation in draining lymph nodes.

### Hypoxia skews T helper cell differentiation by DCs

T cell priming is an essential function of DCs and this process is affected by the TME as well. Immature blood monocyte-derived DCs cultured at hypoxic conditions were found to be biased towards a Th2-stimulatory phenotype by switching to secretion of IL-4 instead of IFN-γ [98]. These T cells mostly stimulate a humoral immune response and suppress DC activation via IL-10 secretion. In addition, DCs cultured under hypoxia secreted increased amounts of osteopontin, which strongly promotes tumor cell migration [98]. However, osteopontin might also promote an immune response, as it promotes IFN-α production via TLR9 signaling in plasmacytoid DCs, which upregulates the expression of MHC-I [113]. The TME also affects T cell recruitment to the tumor, as long-term hypoxia for multiple days was shown to increase the expression cytokines CCL3, CCL5 and CCL20 in mature DCs [114]. These cytokines are chemotactic for both activated and memory T cells, monocytes and immature DCs. Finally HIF-1α activates transcription of retinoic acid receptor-related orphan nuclear receptor gamma (RORγt) and together these two factors regulate the transcription of IL-17 [115]. Therefore, hypoxia increases the differentiation of T cells towards regulatory T helper 17 (T_h_17) cells via IL-6 in a uniquely TGF-β independent fashion [116]. Regulatory T_h_17 cells are associated with host defense and autoimmune inflammatory responses [117, 118] and promote tumor growth [115].

## DISCUSSION

**Figure 2:**
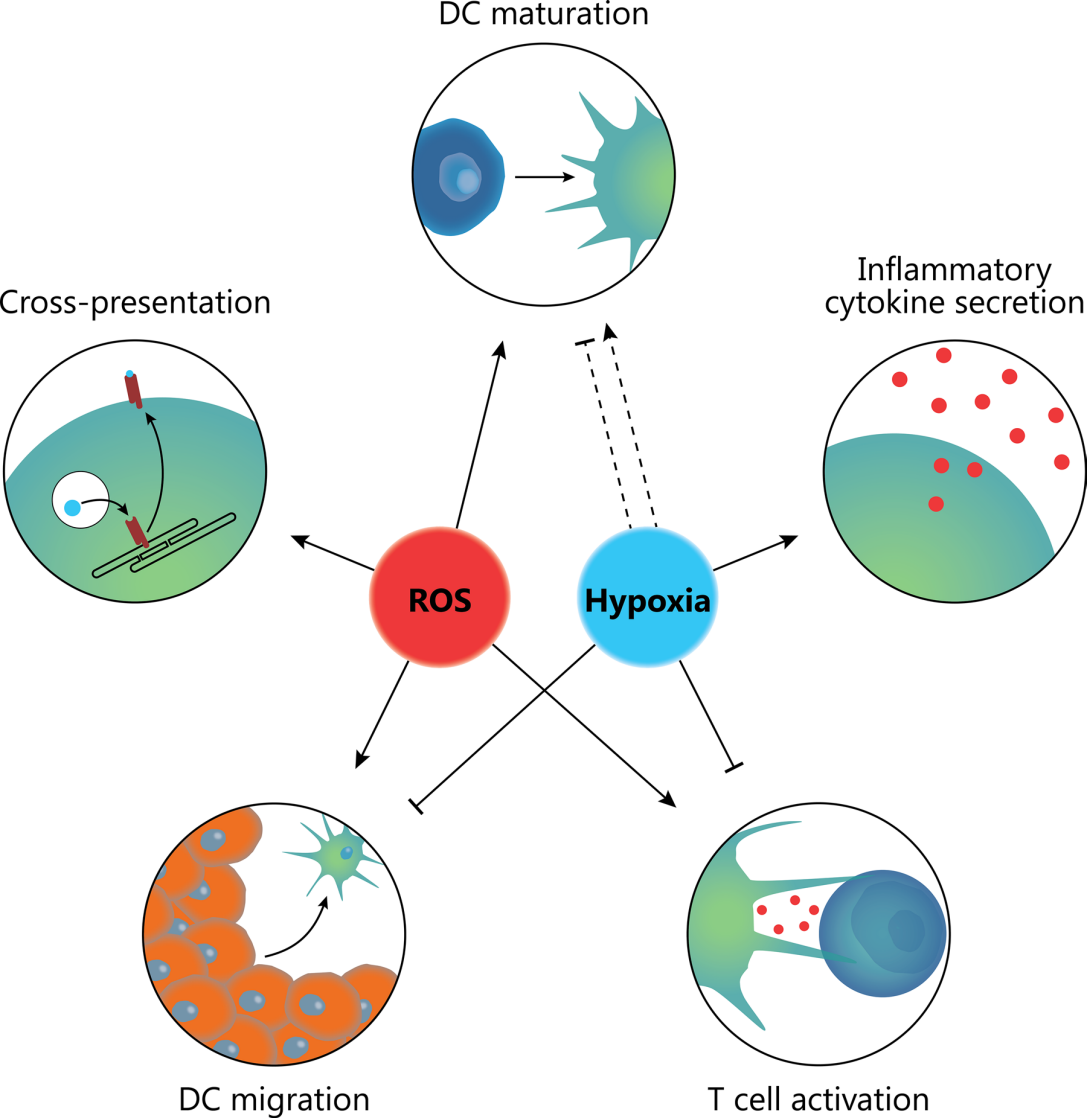
Combined effects of ROS and hypoxia in the TME ROS promote DC maturation, antigen cross-presentation, DC migration and CD8^+^ T cell responses needed for anti-tumor immunity. In contrast, hypoxia can both lower or increase DC maturation and skews T cell responses towards a tolerogenic T_h_17 response. Moreover, hypoxia can inhibit ingress of immature DCs into the tumor whilst blocking migration of mature DCs from the tumor to draining lymph nodes. Hypoxia does however promote secretion of various inflammatory cytokines such as TNF-α.

In this review, we discussed how the hypoxic, oxidative environment of the TME influences invading immune cells. Figure 2 shows an overview of the major effects that have been found so far. The effects of the cytokines and chemokines found in the TME have been quite extensively studied. In contrast, comparatively little is known about the effects on tumor invading immune cells of localized ROS and hypoxia that frequently hallmark the TME. Both the tumor cells and the immune cells produce various forms of ROS, that directly affect the cell physiology but can also target neighboring cells, either by diffusion of ROS or by ROS encapsulated in EVs (Figure 1). ROS generation consumes oxygen and is therefore often paired with local hypoxia, whereas hypoxia can promote formation of ROS, making it difficult to differentiate the effects of ROS and hypoxia from each other. As described in this review, the effects of hypoxia on DC phenotype and maturation are under debate. However, it is becoming increasingly clear that hypoxia stimulates migration of immature DCs but prevents migration of mature DCs. This might result in the TME becoming an immunosuppressive “DC trap”, with limited influx and maturation of immature DCs while the egress of activated DCs is prevented. However, in contrast to this, hypoxia seems to upregulate secretion of various pro-inflammatory anti-tumorigenic cytokines. Maybe these contradicting effects are the result of ROS signaling.

A major question is whether tumor cells use EV release as a protective mechanism from oxidative damage as a result of their high anaerobic metabolic activity, or as an active defense mechanism to prevent immune responses. However, due to the small size and heterogeneity of EVs, it is difficult to study their content. *In vitro* approaches using artificial membranes carrying ROS might help to overcome this problem. Another problem is that resolving the physiological effects of specific sources and types of ROS remains challenging, due to their highly transient nature and the lack of specific probes that offer adequate spatiotemporal resolution. Controlling specific redox signaling and antioxidant pathways would be a valid approach to this problem, since these parameters can be modified with genetic techniques. In addition, ROS can be induced with organellar precision using fusion constructs of proteins with known cellular location with photosensitizer proteins like SuperNova [119]. Likewise, culture media can be supplemented with a wide range of antioxidants or radical-generating systems.

Another key question is whether ROS can be used to treat cancer. A possible avenue would be local administration of pro-oxidants in the TME. Tumor cells often display a defective Nrf2 pathway, rendering them more susceptible to oxidative stress [120], while DC maturation can be enhanced by ROS as described above. In a xenograft mouse model of chronic lymphocyte leukemia, pro-oxidative treatment strongly reduced tumor burden [120]. However, since ROS also has pro-tumorigenic effects, the opposite approach of administrating anti-oxidants is also possible. There have been several randomized controlled trials in which prophylactic effects of such antioxidant supplementation was investigated. However, for incidence of prostate and total cancer in men, supplemental vitamin E had no effects [121–123] and in one study even significantly increased prostate cancer incidence [124].

Since the effects of ROS on cancer and immune cells are complex and dependent on the site of ROS generation and the interplay with hypoxia and immune signaling, targeting ROS by simply administering pro- or antioxidants on might not be sufficient. Targeting ROS or antioxidants to a specific cell type may provide a more successful strategy to combat cancer. For instance, promoting ROS formation in the lumen of endo/phagosomes of DCs could be a strategy to promote antigen cross-presentation [55–57, 60, 61, 63, 125], whereas blockage of mitochondrial ROS formation might increase T cell activation in the lymph nodes [64]. In the paper by Dingjan *et al.*, the photosensitizer protein KillerRed [126] was employed to increase endosomal ROS in DCs by transfecting a plasmid encoding a KillerRed fusion protein targeted to phagosomes. However, transfection of tumor-associated DCs *in vivo* is still very challenging. An alternative approach would be to target DCs with nanoparticles carrying a ROS-inducer [127–129], for example an iron core that promotes generation of highly reactive hydroxyl radicals through Fenton chemistry [130, 131].

In a similar fashion, cancer cells might be specifically targeted with antioxidants to block the pro-tumorigenic effects of ROS. While, as described above, systemic antioxidant therapy proved unsuccessful in cancer, localized interventions are still worth considering. Endosomal NOX2 activity was recently shown to play an important role in progression of prostate cancer [132], which could be targeted (for instance with antibodies) with antioxidant-carrying small particles for exclusive uptake via endocytosis by tumor cells [133]. Another interesting targeting approach is ROS-responsive nanoparticles for targeted delivery of hydrophilic and cationic drugs in ROS-producing cells [134]. In this study, Meng *et al.* showed that MnO_2_-based nanoparticles selectively release the HIF-1 inhibitor acriflavine in tumor cells after oxidation by H_2_O_2_ *in vitro* and in a mouse model of colon cancer. Although the authors did not investigate uptake by phagocytic cells, it is likely that this method is also capable of releasing compounds in phagosomes. Finally, it might be highly beneficial to sequester lipid peroxidation products such as MDA and 4-HNE due to their negative impact on DC function, as described above. Doing so would protect DCs against these effects without interfering with ROS-induced cross-presentation and DC maturation. Several potential compounds have been identified recently that warrant further investigation, of which histidine-containing dipeptides are currently the most promising [135–137].

Given that cancer cells use hypoxia and ROS to reprogram immune and stromal cells in the TME to prevent an immune response and augment tumor progression, while at the same time the immune system uses ROS to signal inflammation and combat infection, ROS have huge therapeutic potential for combating cancer. Given this duality, the timing and localization of pro- or antioxidant interventions is likely highly critical. Understanding the intricate pathways of the production, signalling and effector responses of hypoxia and ROS is essential for designing such therapies.

## Abbreviations

4-HNE: 4-hydroxynonenal
AMPK: 5′ adenosine monophosphate-activated protein kinase
AP-1: activator protein 1
AQP: aquaporin
ASK1: apoptosis signal-regulating kinase 1
CCL: C-C Motif Chemokine Ligand
DC: dendritic cell
EV: extracellular vesicle
HIF: hypoxia-induced factor
IFN: interferon
JNK: c-Jun N-terminal kinase
LFA-1: lymphocyte function-associated antigen 1
MAPK: mitogen-associated protein kinase
NF-κb: nuclear factor kappa B
NOX: NAD(P)H oxidase
Nrf2: Nuclear factor-erythroid 2 p45-related factor 2
Prx: peroxiredoxin
ROS: reactive oxygen species
Siglec-1: sialic-acid-binding immunoglobin lectin 1
SOD: superoxide dismutase
TLR: Toll-like receptor
TME: tumor microenvironment
TNF-α: tumor necrosis factor alpha
Trx: thioredoxin
VEGF: vascular endothelial growth factor
